# Predicting Organization Performance Changes: A Sequential Data-Based Framework

**DOI:** 10.3389/fpsyg.2022.899466

**Published:** 2022-05-18

**Authors:** Meiqi Song, Xiangling Fu, Shan Wang, Zhao Du, Yuanqiu Zhang

**Affiliations:** ^1^Key Laboratory of Trustworthy Distributed Computing and Service (BUPT), Ministry of Education, School of Computer Science (National Pilot Software Engineering School), Beijing University of Posts and Telecommunications, Beijing, China; ^2^Department of Finance and Management Science, University of Saskatchewan, Saskatoon, SK, Canada; ^3^Business School of Sport, Beijing Sport University, Beijing, China

**Keywords:** organization performance changes, business decline, business recovery, risk warning status, a sequential data-based framework, bi-directional long short-term memory (Bi-LSTM), news sentiment

## Abstract

The business environment is increasingly uncertain due to the rapid development of disruptive information technologies, the changing global economy, and the COVID-19 pandemic. This brings great uncertainties to investors to predict the performance changes and risks of companies. This research proposes a sequential data-based framework that aggregates data from multiple sources including both structured and unstructured data to predict the performance changes. It leverages data generated from the early risk warning system in China stock market to measure and predict organization performance changes based on the risk warning status changes of public companies. Different from the models in existing literature that focus on the prediction of risk warning of companies, our framework predicts a portfolio of organization performance changes, including business decline and recovery, thus helping investors to not only predict public company risks, but also discover investment opportunities. By incorporating sequential data, our framework achieves 92.3% macro-F1 value on real-world data from listed companies in China, outperforming other static models.

## 1. Introduction

The sustainability of organization performance is challenged by today's turbulent business environment. Understanding, gaining insights on and being capable of predicting performance changes of organizations under risks, including business decline and recovery (Schendel et al., 1976; Robbins and Pearce, 1992; Pretorius, 2009; Schweizer and Nienhaus, 2017; Barbero et al., 2020), become a critical component of organization intelligence to both managers and investors. Organization performance change prediction serves as an early risk warning for both companies and investors. With accurate predictions, companies can stay prepared for potential failures in the future, and make sound strategic business decisions to catch up the upcoming business opportunities in a business decline with the promise of recovery. Investors also need to make informed decisions on whether to continue supporting a company based on accurate organization performance prediction, as well as discovering business opportunities if a business recovery is predicted. However, such predictions are increasingly difficult, since the business environment is becoming increasingly dynamic, complicated by the rapid development of disruptive information technologies and COVID-19 pandemic (Amankwah-Amoah et al., 2021; Sopha et al., 2021).

Organization performance changes, including business decline and business recovery, has been widely studied, but using machine learning to predict organization performance changes is still in its nascent stage. ***Business decline*** is often studied from the perspective of business failures (Ucbasaran et al., 2013; Sheng and Lan, 2019), which are defined and measured by business discontinuity of new firms (Carter et al., 1997; Ibrahim and Paulson, 2008), organization bankruptcy (Moradi and Beigi, 2020), financial stress and the delisting of public companies in the stock market (Martinez and Serve, 2017). One of the central interests in the business decline research is to study factors that lead to business decline. For example, Schendel et al. (1976) provides a comprehensive summary of reasons for business downturn, including increasing costs, demand declines, decreasing revenues, strikes, increased competitive pressure, management problems and marketing problems. Recent research focuses on a specific aspect of organization management and study their relationship with business failure (Charitou et al., 2007; Chiraz and Anis, 2013). For example, a group of researchers studies the involuntary delisting of public companies and how they are impacted by various aspects of organization management, such as ownership structure (Charitou et al., 2007), the proportion of independent directors, the size of the board, and the quality of audit (Chiraz and Anis, 2013). ***Business*** ***recovery*** is another state of organizational performance change, and is often studied as business turnaround (Pretorius, 2009). Turnaround strategies have been widely studied. For example, Schendel et al. (1976) classified eight types of management actions and environmental events as turnaround strategies, such as organization and management changes, marketing program changes, diversification and efficiency increase, etc. Through a content analysis of the literature, Schweizer and Nienhaus (2017) summarized four categories of turnaround strategies, include operational restructuring, management replacements, portfolio restructuring, and financial restructuring. Barbero et al. (2020) studied whether the speed of retrenchment process contributes to business turnaround.

In the recent years, researchers are increasingly interested in using machine learning to predict both business performance decline and turnaround. The prediction-based research mainly focuses on predicting business failure using the delisting event of public companies from stock exchanges (Hwang et al., 2014; Zhou et al., 2017; Endri et al., 2020). Delisting is the removal of a listed security from a stock exchange. Delisting can be voluntary or involuntary delisting, and involuntary desliting is often considered as business failure, especially involuntary delisting for bankruptcy and other negative reasons (Charitou et al., 2007; Chiraz and Anis, 2013; Martinez and Serve, 2017). Researchers have used various machine learning algorithms and features to predict company delisting. For example, Endri et al. (2020) used support vector machine (SVM) to predict the delisting of Islamic stocks based on financial information. Wang and Campbell (2010) used Altman's Z-score model to predicting the delisitng of of Chinese public companies companies. The recent research in this stream also adds non-financial information in the predictive models (Sheng and Lan, 2019; Wang and Li, 2020; Li et al., 2021). However, the prediction-based research on business recovery or turnaround is less studied.

This research will fulfill this research gap by developing a Sequential Data-based Prediction Framework on Organization Performance Changes (SDP-OPC) to predict organization performance changes, including both business decline and business recovery. More specifically, the research leverages a special mechanism of risk warning in China stock market that are issued to companies under risks, and the listing status with a risk warning label added or removed provides an ideal context to study business decline or business discovery. This SDP-OPC framework aims to predict the listing status changes as a reflection of business decline and recovery of public traded companies in China stock market. To improve the accuracy of the prediction model, the framework combines sequential data from various sources, including both financial and non-financial data such as organization basic demographic information, organization ownership structure, and mass media news sentiment. The framework is further validated based on a quarterly panel data of 243 public companies listed in China stock market. Experiments are conducted to compare the prediction performance of the SDP-OPC framework and other benchmark models such as Random Forest (RF), eXtreme Gradient Boosting (XGBoost), and Light Gradient Boosting Machine (LightGBM) and the SDP-OPC framework demonstrates higher accuracy rates than the benchmark models.

This research makes the following contributions to the literature. ***Firstly***, this research adds to a stream of emerging research on building organization intelligence based on artificial intelligence algorithms to manage a sustainable organization performance (Galbraith and Podhorska, 2021; Suler et al., 2021). ***Secondly***, the SDP-OPC framework proposed in this research predicts a portfolio of organization performance changes, including normal business status, business decline and business recovery. To date, some studies have been conducted to predict business decline or failure as indicated by the delisting, or different risk statuses of companies (Sheng and Lan, 2019; Endri et al., 2020; Wang and Li, 2020; Li et al., 2021). However, to our best knowledge, no prior research has studied the prediction of a portfolio of organization performance changes. ***Thirdly***, the framework is a dynamic model that takes into account the time series data to improve prediction accuracy. To our best knowledge, most prior research used static models such as RF, XGBoost and LightGBM (Endri et al., 2020; Wang and Li, 2020; Li et al., 2021)s. ***Finally***, the model incorporates multiple dimensional features, including both financial and non-financial data to improve prediction accuracy. From a managerial perspective, the framework can be implemented by the organizations and investors to dynamically monitor and predict the performance changes of the listed companies, and make informed management and investment decisions based on such predictions. The findings on the differential importance of features also provide managers suggestions on the measures to take in order to recover from business decline.

The paper is structured as the following. In Section 2, we introduce the research background and prior related work. The models used in the framework and experiments are also introduced in this section. In Section 3, we describe the SDP-OPC framework for predicting organization performance changes. In Section 4, we introduce the experiments that we have conducted to validate the framework, including data collection, experimental settings and goals, and evaluation criteria. The experimental results are explained in Section 5. Finally, We conclude the paper by discussing the major findings, contributions and research limitation and future research in Sections 6, 7.

## 2. Research Background and Related Work

### 2.1. Research Background

This research uses the risk warning mechanism in China stock market to study the prediction of business decline and recovery prediction. In China stock market, two stock exchanges, the Shanghai Stock Exchange (SHSE) and Shenzhen Stock Exchange (SZSE), each has an early warning system to predict and inform investors' companies risks, which are set according to the “Rules Governing the Listing of Stocks (RGLS)” released in July 2012 by the China Security Regulatory Commission. There are two types of risk warnings: indication of the delisting risk and indication of other risks. ***The*** ***delisting risk warning*** is issued to a firm if significant abnormal financial conditions are observed, such as negative audited net profit of the company in the last two consecutive fiscal years, negative audited net worth of the company in the last fiscal year, and severe violations of record disclosure in annual reports (Zhou et al., 2017). According to “Rules Governing the Listing of Stocks (RGLS)” released in July 2012, ***the*** ***other risk warning*** is issued to a company if other risks are detected, such as the main bank account of the company being blocked, adversely affected company operations which are unlikely to be recovered in 3 months, and the board failing to hold meetings on a regular basis and being unable to reach a resolution (Zhou et al., 2017).

Firms subjecting to either delisting risk or other risk warning receive the following special treatment from the stock exchanges. ***First***, stock exchanges will label stocks of companies with delisting risk warning “*ST,” and those with other risk warning “ST.” If a company receives both types of warnings, the stock will be labeled “*ST.” Such labels are made visible to investors to warn them the potential risk of such stocks. In general, “*ST” prefixed stocks (also called *ST stocks hereafter) are considered highly risky, and much riskier than “ST” prefixed stocks (also called ST stocks hereafter). ***Second***, restrictions are put on the transactions of those “ST” or “*ST” labeled stocks. For example, the limit of the price changes (i.e., increase or decrease) within a trading day is set as 5% for a stock with a risk warning and 10% for a normal stock (Zhou et al., 2017). ***Third***, a company is also required to make adjustments to improve their financial conditions and management practices, to be compliant with the regulation. Otherwise, the company runs the risk of being delisted from the stock exchanges. For example, if a *ST labeled company cannot improve its financial conditions in the next year, the company will be forced to enter a delisting consolidation period, resulting in the termination of the listing of the company in the stock exchange.

In December 2020, “Rules Governing the Listing of Stocks (RGLS)” was revised by SHSE and SZSE. The new regulation modifies the conditions for listed companies to be labeled “*ST.” According to the new regulation released in December 2020, the delisting risk warning is issued to a firm if significant abnormal financial conditions are observed, such as negative audited net profit of the company in the last fiscal years, negative audited net worth of the company in the last fiscal year, and severe violations of record disclosure in annual reports. Since the dataset we collected doesn't involve data after December 2020, we only consider the old regulation released in July 2012.

### 2.2. Predicting Organization Performance Changes

Such risk warning mechanism and company labels in China stock market clearly indicate the performance status and health of a company, providing researchers valuable opportunities to study business decline and business recovery. One stream of research in organization performance predictions used the unique context of China stock market to study factors that lead to the business failure and business recovery (Sheng and Lan, 2019). Researchers have tried to use various machine learning or deep-learning methods to predict the business performance change of listed companies based on the special treatment of stock in China stock market. These models mainly use financial information to predict business decline, with the aim to monitor the risks of listed companies through such predictions. Zhou et al. (2017) divided the company's listing status into four groups: the normal companies without any risk warning, the companies with delisting risk warning, the companies with other risk warning and the delisted companies. Then they developed a listing status prediction method that combines C5.0 decision trees with an improved filter feature-selection method. Li et al. (2016) selected 200 A-share listed manufacturing companies as samples and developed a risk assessment model of listed companies to predict their likelihoods of receiving a ST or *ST special treatment based on AdaBoost-SVM. Liu et al. (2010) used not only financial information, but also two features about related party transactions, related-party transactions of conglomerates and ratio of related-party transaction amount to total assets, and applied BP neural network to predict the ST risk status of the enterprise groups.

Researchers also increasingly incorporate texts as a data source to predict business decline. For example, Zhao et al. (2019) proposed a dynamic default probability prediction framework to predict whether the company will be marked with “ST” in the next quarter. Their framework not only considers the financial data, but also utilizes a long short-term memory (LSTM) neural network to obtain the semantic representation of news titles so that the model can alleviate the impact of financial fraud issues. Sheng and Lan (2019) found that volume of mass media news can be used to predict the *ST treatment of the firms, while sentiment has mixed predictive power. In addition to the news texts, some researchers use the texts in the “Management Discussion and Analysis (MD&A)” section of company annual reports to predict whether a company will receive a ST or *ST special treatment. Wang and Li (2020) constructed two textual feature indicators, superficial tone (STONE) and implicit propensity for default (IPD), and trained an eXtreme Gradient Boosting (XGBoost) model based on financial indicators and textual features to predict the ST risk status of listed companies. Li et al. (2021) mined MD&A text from three dimensions (text similarity, text emotional value and text readability) by natural language text analysis technology. Then they tried several methods to build a company early risk warning model, including logistic model, decision trees, support vector machine (SVM) model and neural network, and concluded that the textual information of MD&A improved the prediction accuracy.

To our best knowledge, most of the research on prediction-based organization performance changes focus on business decline (i.e., whether a company will be issued ST treatment or not), and only one has studied business recovery (Zhou, 2013). Zhou (2013) employed the Adaptive Boosting (Adaboost) method to construct a model to predict the removal of special treatment or delisting risk for the listed company in China. However, this research does not consider the nature of sequential data and used only the most recent year data to predict next year's treatment status of companies. It used only 9 financial indicators, ignoring the news and other non-financial information as a valuable data source to predict organization performance change. Our research aims to propose a framework that uses sequential data and non-financial indicators to improve prediction accuracy.

### 2.3. Bi-Directional Long Short-Term Memory and Other Benchmark Encoders

The SDP-OPC framework proposed and tested in this research uses **Bi-directional Long Short-Term Memory (Bi-LSTM)** as the encoder. Bi-LSTM is a **recurrent neural network (RNN)** variant that handles sequential data. RNNs are networks with loops in them, which build connections between units from a directed cycle. In fact, RNNs can be considered as the result of multiple copies of the same neural network structure, and each network passes some information to the next network. This chain-like nature reveals that RNNs can deal with sequences. The formula (1) defines the hidden state of RNNs at time step *t*, where *x*_*t*_ is the input data at time step *t*, *h*_*t*−1_ is the hidden state at time step *t*−1, *h*_*t*_ is the hidden state at time step *t*, *W* and *b* are learnable parameters, *tanh* is the activation function. Specifically, the hidden state at current time comes from the hidden state at previous time and the current input data, which means RNN can memorize the previous information.


(1) $$\begin{array}{r} h_{t}=\tanh\left(W\left[h_{t-1},x_{t}\right]+b\right) \end{array}$$

But RNN has the well-known problem of gradient exploding or vanishing, so **Long Short-Term Memory (LSTM)** (Hochreiter and Schmidhuber, 1997) and **Gated Recurrent Unit (GRU)** (Cho et al., 2014) are proposed to alleviate this problem. LSTM has a similar chain-like structure, but introduces three gates, input, output and forget gates to control the pass of information in the memory cell. The formula (2) defines three gates and the hidden state at time step *t*, where *h*_*t*_ is the hidden state at time step *t*, *f*_*t*_ is the forget gate, *i*_*t*_ is the input gate, *o*_*t*_ is the output gate, *C*_*t*_ is the cell state at time step *t*. The three gates prevent the recursive derivative from going to infinity or zero in the backpropagation process, and therefore avoids the exploding or vanishing gradient problem in the traditional RNN. In order to reduce computing requirements and processing time, GRU further modifies the structure of LSTM and combined three gates into two gates, update gate and reset gate. The formula (3) defines the update gate, reset gate and hidden state of GRU, where *R*_*t*_ is the reset gate, *Z*_*t*_ is the update gate, $\tilde{h_{t}}$ is the candidate hidden state, *h*_*t*_ is the hidden state at time step *t*. The reset gate can be used to discard historical information unrelated to prediction because it controls how the previous hidden state containing the historical information enters the current candidate hidden state. Then the update gate is used to update the current hidden state *h*_*t*_ based on the previous hidden state *h*_*t*−1_ and the current candidate hidden state $\tilde{h_{t}}$. GRU has simpler structure than LSTM, while LSTM is more flexible due to more gates. In general, both LSTM and GRU are commonly used models to deal with sequential data in various fields.


(2) $$\begin{array}{r} \begin{array}{c} f_{t}=\sigma \left(W_{f}\left[h_{t-1},x_{t}\right]+b_{f}\right)\\ i_{t}=\sigma \left(W_{i}\left[h_{t-1},x_{t}\right]+b_{i}\right)\\ C_{t}=f_{t}⊙C_{t-1}+i_{t}⊙\tanh\left(W_{C}\left[h_{t-1},x_{t}\right]+b_{C}\right)\\ o_{t}=\sigma \left(W_{o}\left[h_{t-1},x_{t}\right]+b_{o}\right)\\ h_{t}=o_{t}⊙\tanh\left(C_{t}\right) \end{array} \end{array}$$

(3) $$\begin{array}{r} \begin{array}{c} R_{t}=\sigma \left(W_{r}\left[h_{t-1},x_{t}\right]+b_{r}\right)\\ Z_{t}=\sigma \left(W_{z}\left[h_{t-1},x_{t}\right]+b_{z}\right)\\ \tilde{h_{t}}=\tanh\left(W_{h}\left[R_{t}⊙h_{t-1},x_{t}\right]+b_{h}\right)\\ h_{t}=Z_{t}⊙h_{t-1}+\left(1-Z_{t}\right)⊙\tilde{h_{t}} \end{array} \end{array}$$

However, conventional RNNs such as LSTM and GRU can only process data in a forward sequence. Schuster and Paliwal (1997) further proposed the bi-directional RNN to model the input sequence forward and backward. Nowadays, RNN and its variants are widely applied to make various business predictions. For example, in the field of financial risk management, Zhao et al. (2019) used LSTM to encode the news titles and financial indicators to predict a company's default risk in the next quarter. Babaev et al. (2019) compared the performance of LSTM, GRU, Bi-GRU and Bi-LSTM in predicting credit scores of credit card customers in the banking industry. This research adopts Bi-LSTM as encoder and uses LSTM, GRU and Bi-GRU as benchmark encoders.

### 2.4. Softmax and Other Benchmark Classifiers

This research employs Softmax as the classifier. Softmax regression model is an extension of the logistic regression model to multiclass classification problems, which calculates the probability that a specific observation belongs to each class (Wang et al., 2018). Logistic classifier is modeled by Bernoulli distribution, and only suitable for two-class classification tasks, but Softmax classifier extends the logistic classifier to solve multiclass classification tasks based on Multinomial distribution.

Except for Softmax, Random Forest (RF), eXtreme Gradient Boosting (XGBoost) and Light Gradient Boosting Machine (LightGBM) are three popular ensemble learning methods. They share the same objective of improving the generalization ability and robustness of the base learners by combining the prediction results of multiple base learners. They are used as benchmark classifiers to validate the performance of the proposed SDP-OPC framework.

**Random Forest (RF):** Random Forest based on bagging is a group of un-pruned classification or regression trees (Breiman, 2001). The idea of bagging requires the model randomly selects a part of the samples from the overall samples as the training set, then this process is repeated many times and one base learner can be obtained every time. Finally, the final result depends on these base learners. Random Forest has the similar process, and it uses Classification and Regression Tree (CART) as the base learners. It obtains the final result by voting or averaging the results of multiple weak classifiers, in order to improve the accuracy and generalization of the results.

**eXtreme Gradient Boosting (XGBoost):** eXtreme Gradient Boosting (XGBoost) is a scalable end-to-end machine learning system for tree boosting (Chen and Guestrin, 2016), which is an improvement to the Gradient Boosting Decision Tree (GBDT). Compared with GBDT, XGBoost expands the loss function using the second-order Taylor expansion, which is conducive to make gradient descent faster and support the custom loss function. Besides, XGBoost adds a regularization term based on the number and weights of leaf nodes to control the complexity of the tree so that the model can avoid the overfitting phenomenon.

**Light Gradient Boosting Machine (LightGBM):** Light Gradient Boosting Machine (LightGBM) was proposed by Microsoft to deal with the problems with XGBoost (Ke et al., 2017). XGBoost is time consuming and takes up considerable memory due to the exact greedy algorithm it uses. LightGBM uses a histogram-based decision tree algorithm, a leaf-wise leaf growth strategy with depth restrictions, histogram difference acceleration, and several other techniques to save memory and speed up the computation.

These ensemble learning methods are popular and have been widely used in the field of financial risk management. For example, Wang and Li (2020) constructed two textual features, superficial tone (STONE) and implicit propensity for default (IPD), and trained an XGBoost model based on financial indicators and textual feature indicators to predict the financial default of listed companies. Qian et al. (2022) proposed a heuristic algorithm based on permutation importance (PIMP) for feature selection, compared the prediction performance of RF, XGBoost and LightGBM in the task of financial distress prediction, and confirmed that PIMP can enhance the prediction performance of classifiers by selecting proper features.

## 3. A Sequential Data-Based Prediction Framework on Organization Performance Changes

### 3.1. The SDP-OPC Framework

This research proposes a sequential data-based prediction framework on organization performance change prediction (i.e., the SDP-OPC framework). The goal of the framework is to predict the organization performance change and therefore the risk status change of the listed public companies in the China stock market: ***business continuity, business decline*** and ***business recovery*** (shown in Table 1). Business continuity is considered as a normal status of company performance. If a company receives no risk warning during the prediction period, the company is member of ***business continuity*** class. If a company is implemented as ST or *ST, experiences an implementation change from ST to *ST, enters the delisting consolidation period or is completely delisted, the company is classified as a member of ***business decline*** class. If a company experiences the removal of a ST or *ST warning label, or the change of the risk warning implementation from *ST to ST, the company is considered as a member in the ***business recovery*** class. Sequential data is used in the prediction, and the target is to predict the risk status changes of a specific company during the period between *T* and *T*+2, given the data of a set of features before the time *T*.

**Table 1:** Three categories of listed company status.

**Company status**	**Special treatment of stock**
Business continuity: Normal Status (NS)	Without risk warning implementation during the observation period*
Business decline: Downturn Status (DS)	If the following event happens during the observation period
	∙ Being implemented ST
	∙ Being implemented *ST
	∙ Implementation change from ST to *ST
	∙ Entering the delisting consolidation period or being delisted
Business recovery: Upturn Status (US)	If the following event happens during observation period
	∙ Removal of ‘ST’
	∙ Removal of ‘*ST’
	∙ Implementation change from *ST to ST

*The observation period in the dataset is from Jan 1st, 2020 to June 30, 2020.

**Figure 1:**
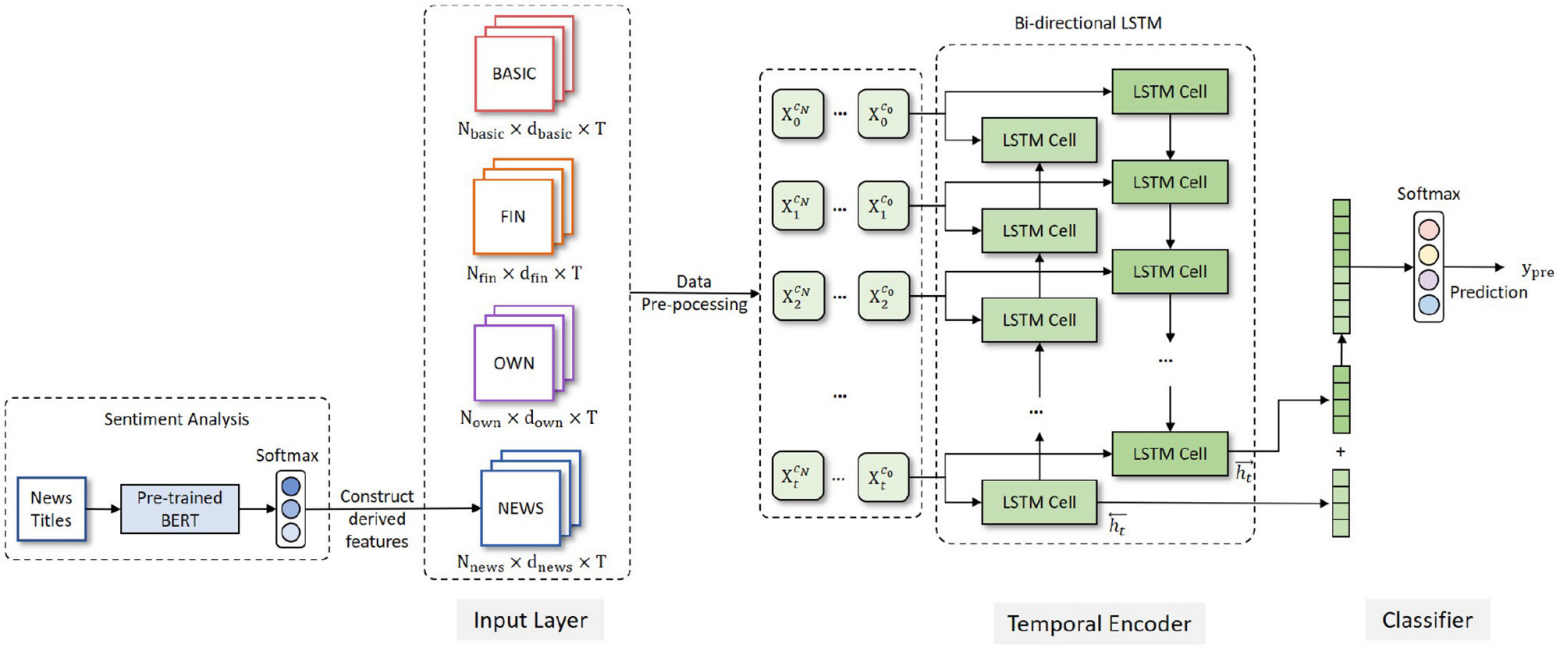
A sequential data-based prediction framework for organization performance changes (SDP-OPC).

As shown in Figure 1, the **SDP-OPC** framework consists of three parts: Input Layer, Temporal Encoder and Classifier.

#### Input Layer

In accordance with the literature, we mainly use financial data in the input layer. However, a company's performance changes cannot solely be interpreted from financial indicators. For certain reasons, the financial reports disclosed by some companies may contain false information, which can adversely impact the prediction performance. Other information, including the ownership structure and public opinions, can make up the prediction inaccuracy caused by financial fraud issues.

In our framework, the input layer includes the raw data about 34 features that fall into 4 categories: company basic information (BASIC), financial indicators (FIN), ownership concentration indicators (OWN), and news sentiment information (NEWS). The features are shown in Table 2.

**Table 2:** Feature indicators of the dataset.

**Categories**	**Features**	
Basic Information (BASIC)	Company survival age (*f*_1_)	
Financial Indicators (FIN)	Per share index	Earnings per share (EPS) (*f*_2_)
		Operating profit per share (RMB/share) (*f*_3_)
		Net assets per share (RMB/share) (*f*_4_)
		Capital reserve per share (RMB/share) (*f*_5_)
	Profitability	Return on net assets (weighted) (%) (*f*_6_)
		Return on assets (%) (*f*_7_)
		Net asset interest rate (TTM) (%) (*f*_8_)
		Return on invested capital (%) (*f*_9_)
		Net profit margin on sales (TTM) (%) (*f*_10_)
		Sales cost rate (%) (*f*_11_)
		Total operating cost/total operating revenue (TTM) (%) (*f*_12_)
	Operation Ability	Turnover rate of accounts receivable (Times) (*f*_14_)
		Inventory turnover rate (Times) (*f*_15_)
		Turnover days of accounts receivable (days/time) (*f*_16_)
		Total asset turnover_ TTM (Times) (*f*_17_)
		Turnover rate of fixed assets (Times) (*f*_18_)
	Solvency	Current ratio (%) (*f*_19_)
		Quick ratio (%) (*f*_20_)
		Net asset liability ratio (%) (*f*_21_)
		Cash ratio (%) (*f*_22_)
	Growth	Growth rate of earnings per share (%) (*f*_23_)
		Growth rate of operating revenue (%) (*f*_24_)
		Net profit growth rate (%) (*f*_25_)
		Growth rate of net assets (%) (*f*_26_)
		Growth rate of total assets relative to the beginning of the year (%) (*f*_27_)
		Growth rate of operating profit (%) (*f*_28_)
Ownership Concentration Indicators (OWN)	Shareholding ratio of the first largest shareholder (*f*_29_)	
	Total shareholding ratio of top 5 shareholders (*f*_30_)	
	Total shareholding ratio of top 10 shareholders (*f*_31_)	
News Sentiment Information (NEWS)	Percentage of positive emotional news of company *c* in the quarter *t* (*f*_32_)	
	Percentage of neutral emotional news of company *c* in the quarter *t* (*f*_33_)	
	Percentage of negative emotional news of company *c* in the quarter *t* (*f*_34_)	

The data for the BASIC, FIN and OWN feature groups can be extracted directly from database, but news sentiment information requires special processing before they enter the input layer. A sentiment analysis component is added before the input layer of the framework to calculate the sentiment scores of the company news titles. A pre-trained Bidirectional Encoder Representation from Transformers (BERT) (Devlin et al., 2019) model (L=12, H=768, A=12) is utilized, which has been pre-trained on a large-scale Chinese news title corpus. We manually labeled more than 3000 news titles of listed companies to fine-tune the BERT model and obtain the semantic representation of every news title. The representation is fed into a Softmax classifier to get the sentiment classification (positive, neutral and negative) of each news title. Then the percentage of different emotional news of every company in every quarter is calculated to obtain the news sentiment information (NEWS) as shown in Table 2. Finally, the news sentiment information (NEWS) is integrated with BASIC, FIN and OWN, which are cleaned and become the input of the encoding layer.

#### Temporal Encoder

**Figure 2:**
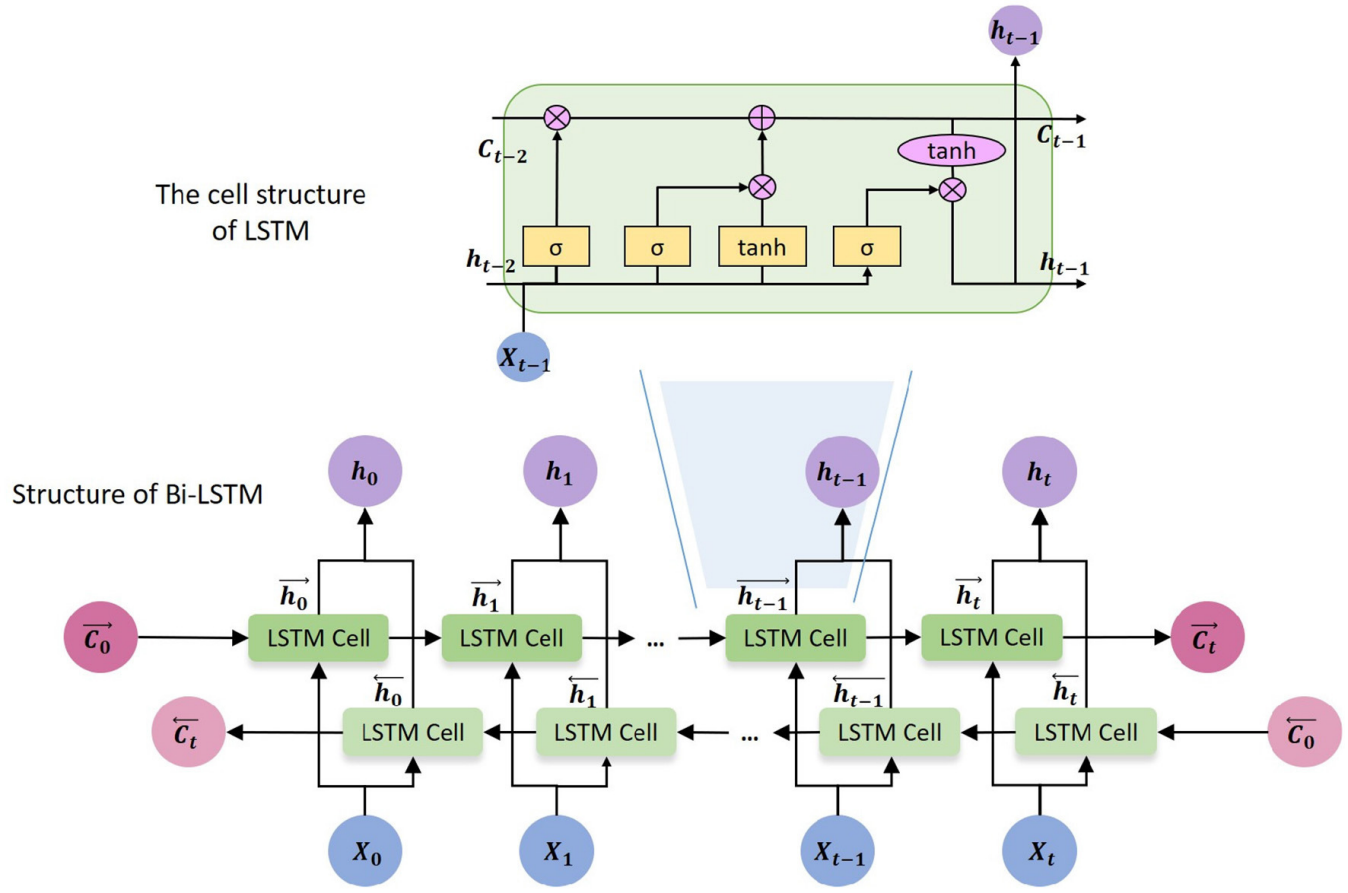
The cell structure of LSTM and Bi-LSTM.

The framework uses Bi-directional Long Short-Term Memory (Bi-LSTM) as encoder. Bi-LSTM is developed based on Long Short-Term Memory (LSTM). The core idea of LSTM (Hochreiter and Schmidhuber, 1997) is that every memory cell has three gates to control the passing of information. LSTM captures long-range dependencies more accurately than previous RNN models, and has good performance in many applications, including risk management. However, the basic LSTM cannot encode information from back to front, which means it can only access the previous information at every specific time step. But Bi-LSTM is able to process the sequential data in both forward and backward directions, and obtain two separate hidden states $\vec{h_{t}}$ and $\overset{⃖}{h_{t}}$. In this work, we use a single layer Bi-LSTM as an encoder which is composed of a forward LSTM and a backward LSTM and is able to capture the inner relation between previous time steps, the current time step and next time steps. The cell structure of LSTM and Bi-LSTM are shown in Figure 2.

The following equations define the corresponding hidden layer function of Bi-LSTM.


(4) $$\begin{array}{l} \vec{f_{t}}=\sigma \left(\vec{W_{f}}\left[\vec{h_{t-1}},\vec{x_{t}}\right]+\vec{b_{f}}\right)\\ \vec{i_{t}}=\sigma \left(\vec{W_{i}}\left[\vec{h_{t-1}},\vec{x_{t}}\right]+\vec{b_{i}}\right)\\ \vec{C_{t}}=\vec{f_{t}}⊙\vec{C_{t-1}}+\vec{i_{t}}⊙\tanh\left(\vec{W_{C}}\left[\vec{h_{t-1}},\vec{x_{t}}\right]+\vec{b_{C}}\right)\\ \vec{o_{t}}=\sigma \left(\vec{W_{o}}\left[\vec{h_{t-1}},\vec{x_{t}}\right]+\vec{b_{o}}\right)\\ \vec{h_{t}}=\vec{o_{t}}⊙\tanh\left(\vec{C_{t}}\right) \end{array}$$

$$\begin{array}{l} \overset{\leftarrow }{f_{t}}=\sigma \left(\overset{\leftarrow }{W_{f}}\left[\overset{\leftarrow }{h_{t-1}},\overset{\leftarrow }{x_{t}}\right]+\overset{\leftarrow }{b_{f}}\right)\\ \overset{\leftarrow }{i_{t}}=\sigma \left(\overset{\leftarrow }{W_{i}}\left[\overset{\leftarrow }{h_{t-1}},\overset{\leftarrow }{x_{t}}\right]+\overset{\leftarrow }{b_{i}}\right)\\ \overset{\leftarrow }{C_{t}}=\overset{\leftarrow }{f_{t}}⊙\overset{\leftarrow }{C_{t-1}}+\overset{\leftarrow }{i_{t}}⊙\tanh\left(\overset{\leftarrow }{W_{C}}\left[\overset{\leftarrow }{h_{t-1}},\overset{\leftarrow }{x_{t}}\right]+\overset{\leftarrow }{b_{C}}\right)\\ \overset{\leftarrow }{o_{t}}=\sigma \left(\overset{\leftarrow }{W_{o}}\left[\overset{\leftarrow }{h_{t-1}},\overset{\leftarrow }{x_{t}}\right]+\overset{\leftarrow }{b_{o}}\right) \end{array}$$

(5) $$\begin{array}{rc} \overset{⃖}{h_{t}} & =\overset{⃖}{o_{t}}⊙\tanh\left(\overset{⃖}{C_{t}}\right) \end{array}$$

(6) $$\begin{array}{rc} h_{t}^{c} & =\vec{h_{t}}⊕\overset{⃖}{h_{t}} \end{array}$$

In formulas (4)(5)(6), → and ← denote the forward and backward process, respectively. *W* and *b* are model parameters that are learned during the model training process. *f*_*t*_, *i*_*t*_, *o*_*t*_ are the forget gate, input gate and output gate of LSTM cells, respectively. *C*_*t*_ is the cell state at time step *t*. The hidden state from the last time step, which is the concatenated vector of $\vec{h_{t}}$ and $\overset{⃖}{h_{t}}$, is used as the representation of the listed company *c*, as shown in formula (6).

#### Classifier

The output of the encoder is then passed to the Softmax classifier to obtain the probabilities *y*_*prob*_ of different classes, which are then converted to the integer value *y*_*pre*_. *y*_*pre*_ indicates the predicted class of a specific company and is the final output of the SDP-OPC framework.

### 3.2. Data Preprocessing

The framework also includes a data preprocessing component, and only cleaned data in the input layer are encoded and analyzed. The data preprocessing process is illustrated in Algorithm 1, and explained in the following.

**Algorithm 1:** DataPre Algorithm

**Input**: Raw Data(BASIC $\in {ℛ}^{N_{basic}\times d_{basic}\times T}$, FIN $\in {ℛ}^{N_{fin}\times d_{fin}\times T}$, OWN $\in {ℛ}^{N_{basic}\times d_{basic}\times T}$, NEWS $\in {ℛ}^{N_{basic}\times d_{basic}\times T}$) and period *T*.
**Output**: Final data and labels *Y*.
1: DataList ← Integrate(BASIC, FIN, OWN and NEWS).
2: **for** *c* in DataList[*id*] **do**
3: recordList=DataList[*id*=*c*];
4: **if** allZero(recordList[BASIC]) or allZero(recordList[FIN] or Num(DataList)!=T+1) **then**
5: Delete recordList;
6: Delete *y*[*id*=*c*];
7: **end if**
8: **end for**
9: **for** *q* in RowNum(DataList)(step=*T*) **do**
10: **for** *i* in ColumnNum(DataList[FIN,OWN]) **do**
11: *x* ← DataList[row=*q*][col=*i*];
12: **if** *x*=Nan **then**
13: *x* ← Mean(between DataList[row=*q*][col=*i*] and DataList[row=*q*+*T*-1][col=*i*]);
14: **end if**
15: **end for**
16: **end for**
17: DataList ← fillUseZero(DataList[NEWS]).
18: Data ← Standarize(DataList);
19: *Y* ← Onehot(*y*).

#### Merging Data

The raw data sources for different categories of features are scattered, so we first need to merge the data from different sources by company id.

#### Handling Missing Values

Some features contain missing values, which can adversely impact the prediction accuracy. We use the following rules to handle the missing values. First, those companies whose basic information is null or all of the financial indicators are null are filtered out. Second, for partially missing financial indicators and ownership concentration indicators, we use the mean value to fill the missing values. Considering the difference in company's business scales, the mean value used to fill the vacancy is calculated using the non-missing values of the corresponding company rather than all companies. Formula (7) shows the calculation of missing values.


(7) $$\begin{array}{r} x_{nan_{iq}}^{c}=\frac{1}{m}\underset{t}{\sum }x_{it}^{c},0\leq t,q\leq T \end{array}$$

where $x_{nan_{iq}}^{c}$ indicates whether the *i*_*th*_ feature's value of company *c* in the *q*_*th*_ quarter is missing; $x_{it}^{c}$ indicates whether the *i*_*th*_ feature's value of company *c* in the *t*_*th*_ quarter is not missing; *m* represents the number of quarters where the *i*_*th*_ feature's value of company *c* is not missing.

Third, for news sentiment information, we use zero to fill the missing values since the missing values indicate that the company has no relevant news in that quarter.

#### Standardizing Data

In order to eliminate the classification bias caused by the unit and scale differences among features, we normalize the data for each feature into a set of values with a mean value of 0 and variance of 1.

#### One-Hot Encoding

The objective variable *y* (i.e., organization performance change) has three classes (shown in Table 1), and has the class label of 0, 1, and 2. *y* = 0 if the company has a normal status (NS); *y* = 1 if the company has an upturn status (US); *y* = 2 if the company has a downturn status (DS). Since *y* is a categorical variable, it is encoded and converted to binary vectors. For example, *y* = 0 is converted to [1 0 0].

## 4. Experiments

### 4.1. Data

In this research, we choose listed companies as the research subjects. On the one hand, the data of listed companies in China are more available than that of unlisted companies or organizations. On the other hand, listed companies in China cover all the major industries, such as manufacturing, information technology, real estate and so on. Therefore, it is more appropriate to use listed companies as samples.

We collected the temporal data on 31 features (shown in Table 2) on the company basic information (1 feature), financial indicators (27 features), ownership concentration (3 features) of all the listed companies in China from January 1, 2017 to December 31, 2019 from the RESSET database (www.resset.com). It is a quarterly panel data of 12 periods (i.e., quarters). Most of the features use the original data downloaded without transformation, except for company survival age, which is calculated as the difference in year between the time of each record and the company launch date.

We also downloaded all the news titles of all the listed companies from the RESSET database from January 1, 2017 to December 31, 2019. The sentiment of new titles is used as a proxy measure of the sentiment of the news because the title summarizes the main content and the sentiment of the news articles, and a focus on new titles can alleviate the computational capacity requirements for processing large number of full texts. 148,803 unstructured news titles were downloaded. The news titles were then processed by the BERT model as shown in Figure 1 to obtain the sentiment score of each news title. The maximum sequence length of BERT model was set to 128. The training batch size, learning rate and training epochs of BERT model were set to 32, 2e-5 and 3, respectively. The sentiment scores of news titles were then aggregated for each company and for each quarter to obtain the following three sentiment scores.

- Percentage of positive news: the ratio of the number of positive news of company *c* in quarter *t* to the total number of news of company *c* in quarter *t*.
- Percentage of neutral news: the ratio of the number of neutral news of company *c* in quarter *t* to the total number of news of company *c* in quarter *t*.
- Percentage of negative news: the ratio of the number of negative news of company *c* in quarter *t* to the total number of news of company *c* in quarter *t*.

We then followed the process shown in Algorithm 1 to pre-process and clean data, including handling missing values, standardize data, and one-hot encoding. Table 3 shows the sample size after data pre-processing. The resulting dataset is strongly imbalanced, with 3,169 companies in the NS class, 35 companies in the US class and 103 companies in the DS class. The strongly imbalanced samples will bias the prediction toward the category with more samples and ultimately affect the accuracy of the prediction (Shi and Wang, 2021). We followed the guideline of under sampling method (Liu and Zhang, 2019) to maintain the samples in each class with a ratio of 3:1:3. According to this ratio, we retained all the samples in the US and DS class, and randomly selected 105 companies from the NS class.

**Table 3:** Sample size before and after under-sampling.

	**Data after pre-processing**		**Data after under-sampling**	
**Class**	**Number of companies**	**Sample size**	**Number of companies**	**Sample size**
NS	3,169	38,028	105	1,260
US	35	420	35	420
DS	103	1,236	103	1,236
Total	3,307	39,684	243	2,916

Additionally, we collected the special treatment of stocks from January 1, 2020 to June 30, 2020 from the RESSET database. Each company is assigned a class label *y* as shown in Table 1: *y* = 0 if the company has a normal status (NS); *y* = 1 if the company has an upturn status (US); *y* = 2 if the company has a downturn status (DS).

### 4.2. Experimental Settings and Goals

We implemented the SDP-OPC framework on RTX 2080 Ti GPU. In our experiments, 70% of the data is assigned to the training set and 30% to the test set. Because the work is considered as a three-class classification task, we choose cross-entropy as the loss function, which is shown as formula (8).


(8) $$L=\frac{1}{N}\sum_{i=1}^{N}L_{i}=-\frac{1}{N}\sum_{i=1}^{N}\sum_{cl=1}^{M}y_{cl}^{i}\log\left(p_{cl}^{i}\right)$$

where *N* is the number of samples, *M* is the number of classes (*M* = 3), $y_{cl}^{i}$ presents whether the real class of sample *i* belongs to the class *cl* or not ($y_{cl}^{i}$ = 1 or 0), $p_{cl}^{i}$ presents the prediction probability that the sample *i* belongs to the class *cl*. In addition, we use Adam optimizer in the training process to make the convergence faster. Besides, we use macro-precision (macro-P), macro-recall (macro-R) and macro-F1 as the evaluation criteria.

The objectives of the experiments are to answer the following questions:
Q1: How do different temporal encoders affect the performance of SDP-OPC?
Q2: How does SDP-OPC perform compared with models without considering temporal data?
Q3: How do different features affect the performance of SDP-OPC?
Q4: How does the importance of features differ in SDP-OPC?

### 4.3. Evaluation Criteria

This research uses Macro-precision (Macro-P), macro-recall (Macro-R) and macro-F1 to measure the prediction performance of models. Precision (P), recall (R) and F1-score are evaluation metrics commonly used for binary classification task. Every binary classification task has a corresponding confusion matrix shown in Table 4, which is used to summarize the prediction results of classification models in machine learning. Confusion matrix whose name comes from the fact that it can easily indicate whether some classes are confused (that is, one class is predicted to be another class), describes the relationship between the actual class and the predicted class in the form of matrix. The confusion matrix of binary classification shown in Table 4 is composed of true positives (TP) and true negatives (TN), false positives (FP) and false negatives (FN). TP means that the sample is predicted to be positive class and the actual class is also the positive class. TN means that the sample is predicted to be negative class and the actual class is also the negative class. FP means that the sample is predicted to be positive class but the actual class is the negative class. FN means that the sample is predicted to be negative class but the actual class is the positive class. Precision(P), recall(R) and F1-score can be calculated based on TP, TN, FP and FN, which is shown in formula (9). The precision is the ability of the model not to label as positive a sample that is negative, the recall is the ability of the model to find all the positive samples and the F1-score is the a weighted average of precision and recall.

**Table 4:** Confusion matrix.

**Actual class**	**Prediction results**	
	**Positive class**	**Negative class**
Positive class	TP	FN
Negative class	FP	TN


(9) $$\begin{array}{r} \begin{array}{cc} P & =\frac{TP}{\left(TP+FP\right)}\\ R & =\frac{TP}{\left(TP+FN\right)}\\ F1 & =2*\frac{P*R}{P+R} \end{array} \end{array}$$

A multi-class classification task can be viewed as multiple binary classification tasks, and its precision and recall metrics can be calculated based on precisions and recalls of the binary classification tasks. Macro-precision (Macro-P), macro-recall (Macro-R) and macro-F1 are used to evaluate the multi-class classification model performance, which are calculated according to formulas (10). *P*_*cl*_, *R*_*cl*_ are the precision and recall for class *cl*. *M* means the number of classes, and *M*=3 in this work.


(10) $$\begin{array}{l} macro-P=\frac{1}{M}\sum_{cl=1}^{M}P_{cl}\\ macro-R=\frac{1}{M}\sum_{cl=1}^{M}R_{cl}\\ macro-F1=2\times \frac{macro-P\times macro-R}{macro-P+macro-R} \end{array}$$

## 5. Experimental Results

### 5.1. Temporal Encoder Architecture Selection (Q1)

To test the performance of the temporal encoder based on Bi-LSTM, we conducted experiments to compare the performance of Bi-LSTM and other RNN variants including LSTM, GRU and Bi-GRU. To improve the performance of the model, we selected the following hyperparameters for each encoder. The learning rates of LSTM, GRU, Bi-GRU and Bi-LSTM are set as 1e-3, 1e-3, 1e-3 and 5e-4, respectively, the hidden units of all these architectures are set to 128, the time step is set to 12, which corresponds to the number of time periods (i.e., quarter), and the batch size is set to 128.

The results shown in Table 5 indicate that our proposed framework using Bi-LSTM as the encoder has better performance than other benchmark encoders. Compared with GRU, the macro-P, macro-R and macro-F1 of our framework are improved by 7.0, 6.7, and 6.8%, respectively. Bi-GRU has the same macro-R as Bi-LSTM, but the macro-P and macro-F1 are much lower than those of Bi-LSTM. Additionally, the bi-directional networks, including Bi-GRU and Bi-LSTM, have better performance than the corresponding unidirectional networks (i.e., GRU and LSTM). The main reason is that the bi-directional networks process sequential data in two opposite directions, which improves the ability of the encoder to mine the changing trend in temporal data.

**Table 5:** Comparison of different temporal encoders.

**Temporal Encoders**	**Macro-P**	**Macro-R**	**Macro-F1**
LSTM	0.911	0.833	0.871
GRU	0.877	0.833	0.855
Bi-GRU	0.886	0.900	0.893
**Bi-LSTM**	**0.947**	**0.900**	**0.923**

*The bold value indicates maximum value in the column. If there are multiple maximum values and the result of SDP-OPC is also the maximum, only the results produced by SDP-OPC will be bold values*.

We further compared the prediction performance using different temporal encoders in different classes of company performance change statuses. The results shown in Table 6 demonstrate that the model using Bi-LSTM as temporal encoder is superior to other temporal encoders in most evaluation criteria. The model with Bi-LSTM as temporal encoder outperforms other temporal encoders for the class NS. For the class US, although the recall rate of Bi-GRU is 0.900 which is higher than Bi-LSTM, the precision rate and F1-score are higher than Bi-GRU. For the class DS, the result is the same.

**Table 6:** The encoder prediction performance by different company statuses.

**Models**	**Normal Status (NS)**			**Upturn Status (US)**			**Downturn Status (DS)**		
	**P**	**R**	**F1**	**P**	**R**	**F1**	**P**	**R**	**F1**
LSTM	0.811	1.000	0.896	1.000	0.700	0.824	0.923	0.800	0.857
GRU	0.833	1.000	0.909	0.875	0.700	0.778	0.923	0.800	0.857
Bi-GRU	0.879	0.967	0.921	0.818	**0.900**	0.857	**0.962**	0.833	0.893
**Bi-LSTM**	**0.909**	**1.000**	**0.952**	**1.000**	0.800	**0.889**	0.931	**0.900**	**0.915**

*The bold value indicates maximum value in the column. If there are multiple maximum values and the result of SDP-OPC is also the maximum, only the results produced by SDP-OPC will be bold values*.

### 5.2. Validation on the Effectiveness of Considering Sequential Data (Q2)

To validate whether using the company's temporal data as model input is useful, we conducted experiments to compare between the SDP-OPC framework and three popular machine-learning models: Random Forest (RF), XGBoost and LightGBM. All of these machine-learning models are based on ensemble learning methods, whose main objective is to improve the generalization ability and robustness of the base learner by combining the prediction results of multiple base learners.

Since the information at the most recent time period usually has the greatest impact on the company's future status, we selected the data in the last period (quarter) as the input of those three benchmark models. Thus, in our experiment, RF, XGBoost and LightGBM are static models. To improve the performance of the model, we set the learning rate of the three benchmark models as 0.01, 0.01 and 0.01, respectively.

The results reported in Table 7 demonstrate that the prediction performance of SDP-OPC is obviously better than that of the three static models. LightGBM has the best performance among the three static models, but the macro-P, macro-R and macro-F1 are still 5.9, 3.3, and 4.6%, respectively, much lower than those of SDP-OPC. To further understand the impact of using sequential data, we compared the prediction performance of different models in different classes of company performance change statuses. As evident from Table 8, the SDP-OPC framework performs much better than other static models, except that LightGBM achieves the same prediction performance as SDP-OPC for the class DS. The maximum difference of precision or recall between SDP-OPC, RF, XGBoost and LightGBM is even more than 10%. So we conclude that SDP-OPC outperforms those static models by using the temporal company data to capture the changing trend of companies.

**Table 7:** Comparison of SDP-ODC and benchmark models.

**Models**	**Macro-P**	**Macro-R**	**Macro-F1**
RF	0.876	0.865	0.866
XGBoost	0.862	0.822	0.841
LightGBM	0.888	0.867	0.877
**SDP-OPC**	**0.947**	**0.900**	**0.923**

*The bold value indicates maximum value in the column. If there are multiple maximum values and the result of SDP-OPC is also the maximum, only the results produced by SDP-OPC will be bold values*.

**Table 8:** The classifier prediction performance by different company statuses.

**Models**	**Normal Status (NS)**			**Upturn Status (US)**			**Downturn Status (DS)**		
	**P**	**R**	**F1**	**P**	**R**	**F1**	**P**	**R**	**F1**
RF	0.839	0.867	0.852	0.889	0.800	0.842	0.900	0.900	0.900
XGBoost	0.839	0.867	0.852	0.875	0.700	0.778	0.871	0.900	0.885
LightGBM	0.844	0.900	0.871	0.889	0.800	0.842	0.931	0.900	0.915
**SDP-OPC**	**0.909**	**1.000**	**0.952**	**1.000**	**0.800**	**0.889**	**0.931**	**0.900**	**0.915**

*The bold value indicates maximum value in the column. If there are multiple maximum values and the result of SDP-OPC is also the maximum, only the results produced by SDP-OPC will be bold values*.

### 5.3. Impact of Different Features on the Prediction Performance (Q3)

We conducted experiments on different combinations of features to understand whether the prediction performance of SDP-OPC varies with the feature combination. In addition, we added a set of two features related to the executive information (EXE), including the chairperson's age and education (denoted as *f*_35_ and *f*_36_, respectively). The chairperson's age is measured by the difference in year between the time of data record and the birthdate of the chairperson, and education is measured by the category of chairperson's education such as Ph.D., Master, Bachelor and others.

We tested seven combinations of features as shown in the first column of Table 9. Table 9 shows that using the combination of BASIC+FIN+NEWS+OWN as model inputs can achieve the best performance on predicting and evaluating company performance changes. Prior research about risk prediction mainly focuses on the financial indicators of companies (Hwang et al., 2014; Endri et al., 2020). However, as shown in our experiments, a model using only financial indicators does not achieve excellent performance, for which the macro-F1 is only 0.868. This is probably because the inclusion of textual information can help to alleviate the bias caused by potential financial fraud.

**Table 9:** Comparison of different feature combinations.

**Feature combinations**	**Macro-P**	**Macro-R**	**Macro-F1**
*FIN*	0.893	0.844	0.868
*BASIC*+*FIN*	0.858	0.856	0.857
*BASIC*+*FIN*+*NEWS*	0.915	0.867	0.890
*BASIC*+*FIN*+*OWN*	0.915	0.867	0.890
*BASIC*+*FIN*+*EXE*	0.800	0.756	0.777
***BASIC*** **+** ***FIN*** **+NEWS+OWN**	**0.947**	**0.900**	**0.923**
*BASIC*+*FIN*+*NEWS*+*OWN*+*EXE*	0.869	0.811	0.839

*The bold value indicates maximum value in the column. If there are multiple maximum values and the result of SDP-OPC is also the maximum, only the results produced by SDP-OPC will be bold values*.

We also tested the framework performance if we add chairperson's information (EXE). Intuitively, the education and age of the chairperson can reflect the experience and qualifications of a company's leaders, which may considerably affect the company's operations, strategies, capabilities and financial conditions. However, according to the experimental results shown in Table 9, the addition of executive information doesn't improve the prediction performance. The macro-F1 for the combination BASIC+FIN+EXE decreases by 8.0%, and the macro-F1 of the combination BASIC+FIN+NEWS+OWN+EXE decreases by 8.4%.

**Figure 3:**
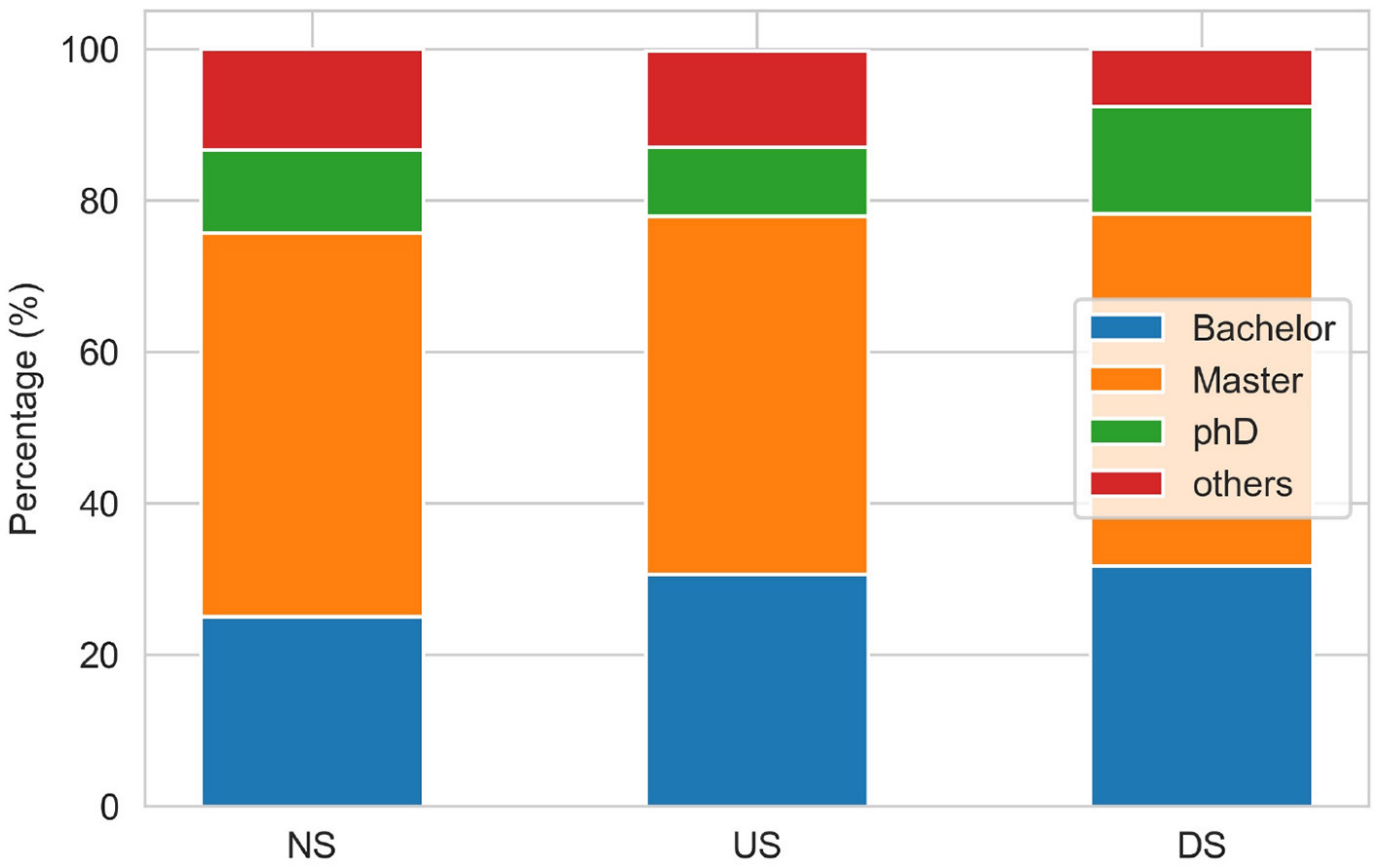
Distribution of chairperson education across different company performance statuses.

**Figure 4:**
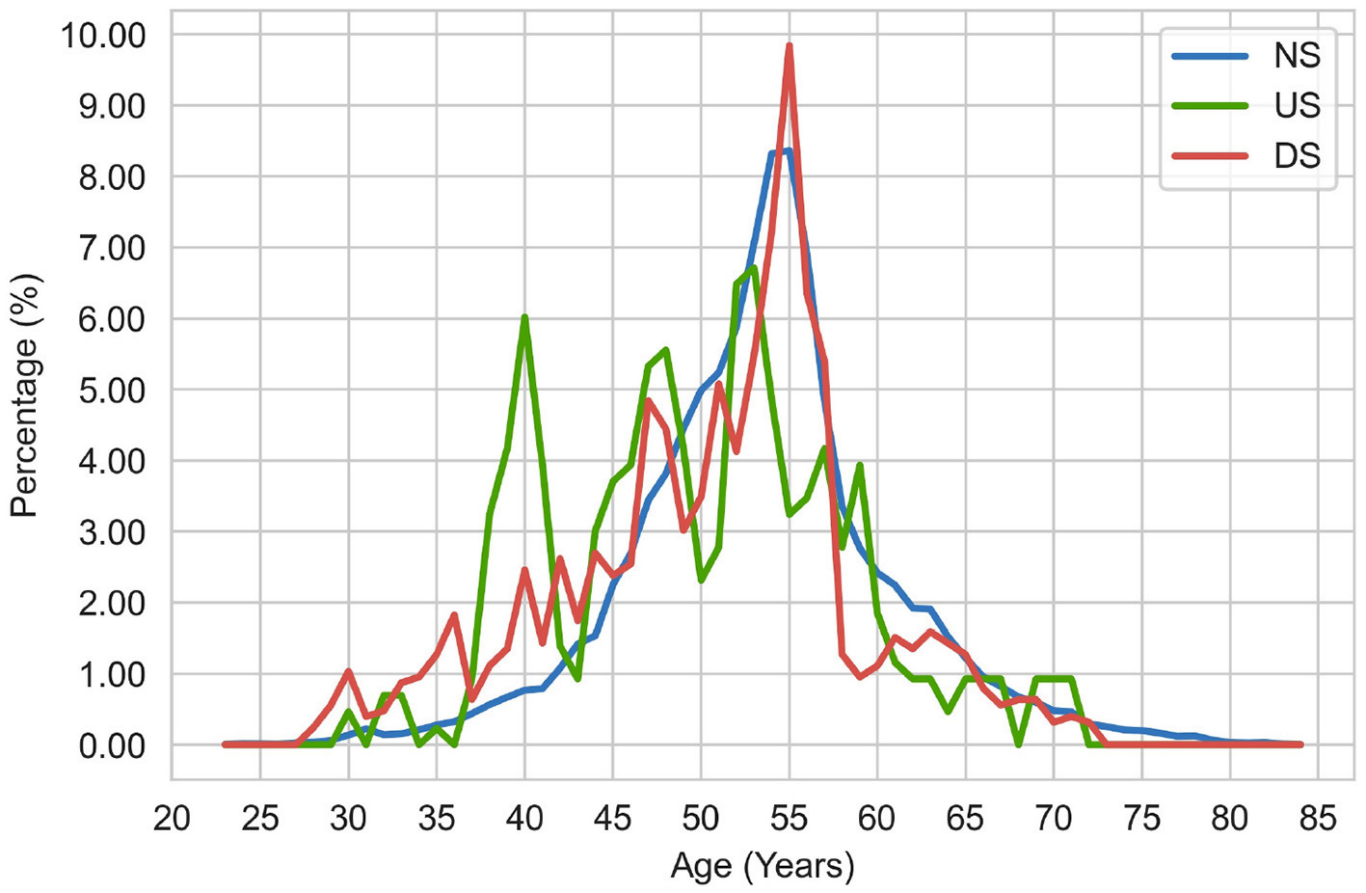
Distribution of chairperson age across different company performance statuses.

To explore the potential reasons, we analyzed the distribution of executive education (see Figure 3) and age across different performance change classes (See Figure 4). As shown in Figure 3, the distributions of company chairperson's education across different performance statuses (NS, US, DS) are very similar. Regardless of the company status, the chairperson's education mainly concentrates on Master or Bachelor degree, which account for more than 75% of the samples. Besides, from Figure 4, we found that peaks of three distribution curves overlap. For companies in the stable performance category, the peak is around 55. For companies in the ascending status category, the age of chairperson is relatively scattered, with several peaks around the age of 40, 48, 53, and 58. And for companies in the descending status category, the age of the chairperson is mainly between 47 and 57, with a peak around 55. Despite these subtle differences, we do not observe much variations in the distributions of chairperson age and education across different classes. Such a pattern may disturb the ability of model to learn from the features, which account for why the personal characteristics of executives of listed companies do not contribute to the prediction of company performance changes. This finding is also consistent with that of Lu's research conclusion (Lu et al., 2020).

### 5.4. Importance of Different Features (Q4)

As shown in Section 5.3, different feature combinations have different prediction performances. To further explore the importance of different categories of features, we computed the feature importance using the idea of permutation importance as shown in Algorithm 2. Permutation importance is measured by the increase in the prediction error of the model after we permuted the feature values. After the feature values are shuffled, the greater the error of the model, the higher the importance of this feature, which means the model depends on this feature for prediction.

**Algorithm 2:** Algorithm of computing feature importance based on permutation importance

**Input**: Test Dataset TD, the number of features *N*, the number of repetitions *M*.
**Output**: Importance of every feature ImportanceList.
1: SDP-OPC ← Load the model that has been trained-well.
2: ImportanceList ← Create an empty list.
3: **for** *n* from 0 to *N*−1 **do**
4: LossSum=0;
5: **for** *m* from 0 to *M*−1 **do**
6: featureCol = TD[col=*m*]
7: TD[col=*m*] ← Shuffle(TD[col=*m*])
8: loss ← Compute the loss value after testing by SDP-OPC model
9: LossSum=LossSum+loss
10: TD[col=*m*]=featureCol
11: **end for**
12: importance=LossSum/*M*
13: ImportanceList.append(importance)
14: **end for**
15: maxImportance = max(ImportanceList)
16: minImportance = min(ImportanceList)
17: **for** *i* in ImportanceList **do**
18: i=(i-minImportance)/(maxImportance-minImportance)
19: **end for**

**Figure 5:**
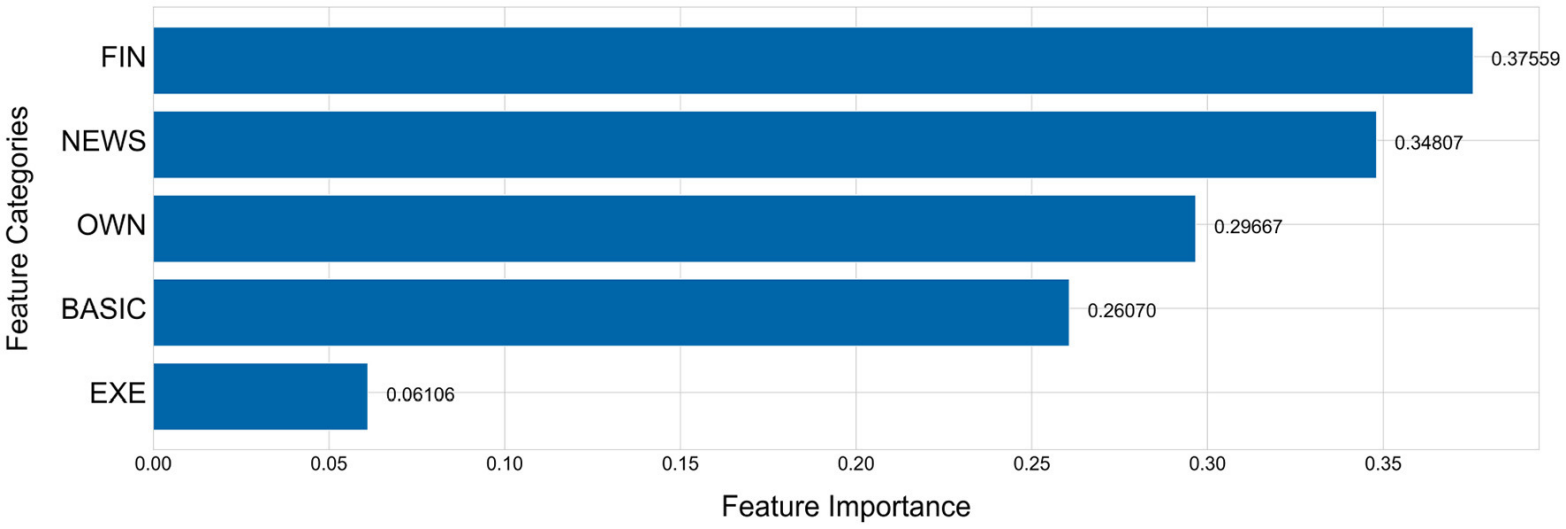
Feature importance of every feature category.

**Figure 6:**
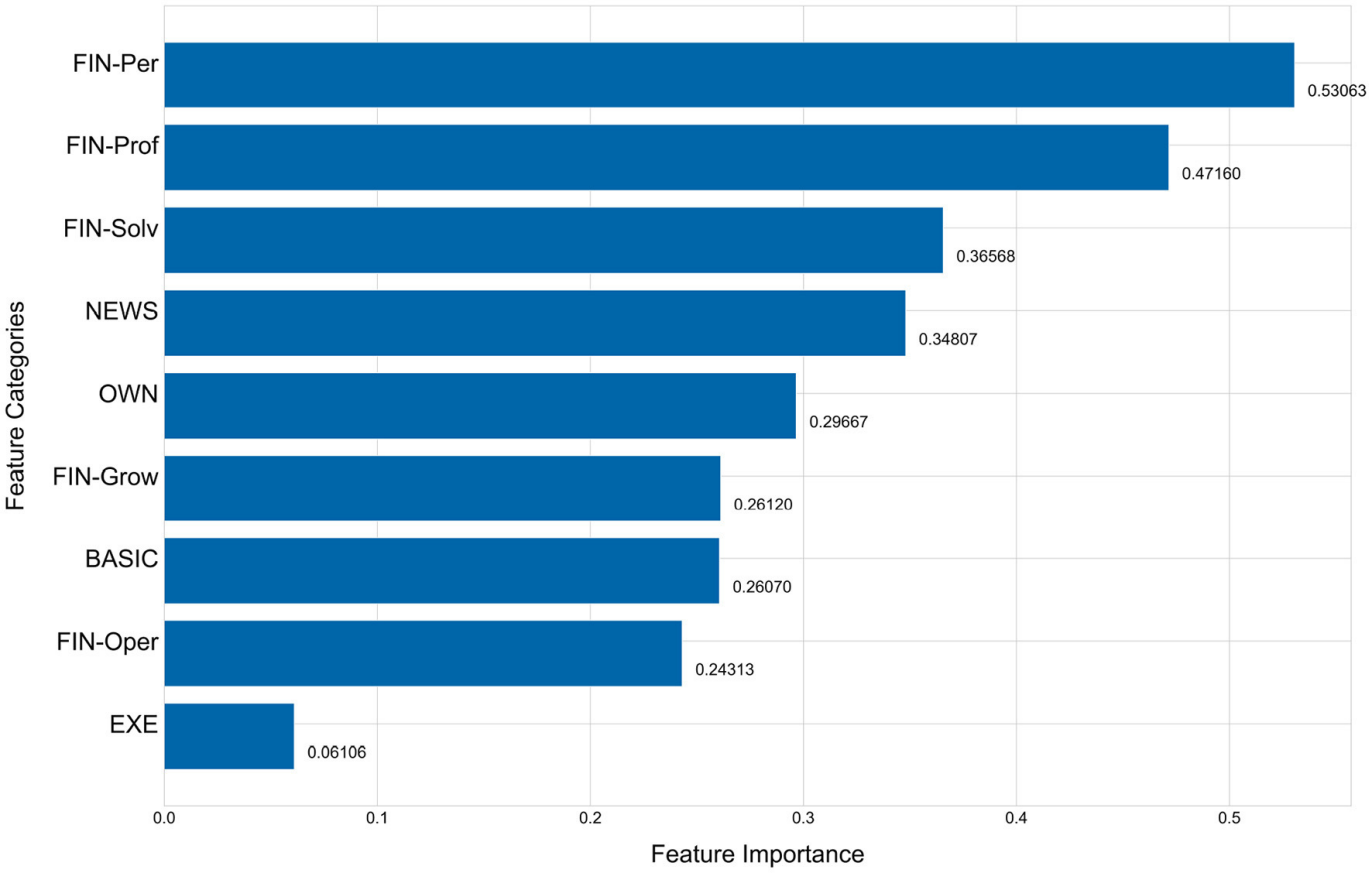
Feature importance of every feature category-A breakdown of the FIN category.

The Figures 5, 6 show the importance of each feature category. Figure 5 shows the importance of features with FIN as one category and Figure 6 breaks the FIN category into 5 sub-categories. The importance of each feature category is calculated by the average of the importance of all features of this category. As evident from Figure 5, the financial indicators (FIN) are the most important for predicting the performance changes of listed companies, followed by news indicators and equity concentration indicators, while the chairperson information has the least importance, which is consistent with the conclusion obtained in Section 5.3. Although the financial indicators have the highest importance, sub-categories are not equally important. As shown in Figure 6, among the five sub-categories of financial indicators (FIN), the sub-category of per share index is higher than other sub-categories, and the category of operation ability is the least important for predicting the company performance changes among all five sub-categories.

## 6. Discussion

### 6.1. Discussion of Major Findings

This research proposes and validates the SDP-OPC framework to predict organization performance changes. The framework incorporates data of various formats to predict a portfolio of performance changes of organizations under risks, including both business decline and business recovery. It includes a custom data pre-processing algorithm, Bi-LSTM as encoder to process time series data and Softmax as classifier to predict the objective performance change variable with multiple categories. The validation of the framework based on a sample of 2,916 observations of 243 companies over 12 quarters indicates that the SDP-OPC framework outperforms other benchmark models such as RF, XGBoost and LightGBM. The maximum difference of precision or recall between SDP-OPC, RF, XGBoost and LightGBM can reach 10%, indicating a significant improvement of the proposed SDP-OPC framework over other benchmark models used in the prior literature (Wang and Li, 2020; Li et al., 2021).

This research also reports the importance of different groups of features in predicting organization performance changes. The experiment results indicate that financial features are the most important one that predicts the organizational performance change. The strong predictive power is also widely observed in other research (Liu et al., 2010; Li et al., 2016; Zhou et al., 2017). Among all the financial features, those related to short-term financial restructuring such as per share index, profitability and solvency have higher predictive power than those related to long-term operational adjustments and growth such as operational ability and growth. The experiment results also show the strong predictive power of mass media news sentiment, next to financial features. This is consistent with Zhao et al. (2019) who found the importance of news sentiment in predict whether the company will be marked with ST in the next quarter, but partially inconsistent with Sheng and Lan (2019) who found that news sentiment has mixed predictive power in predicting the *ST treatment of the firms. Sheng and Lan (2019) found that news sentiment is significantly unfavorable to *ST firms compared to non-*ST firms, but there is no significant difference in news sentiment between delisted *ST firms and other *ST firms. Such a difference can be explained by the slightly different objective variables we use. Our objective variables focus on the general categories of business decline and business recovery, but Sheng and Lan (2019) focus on only studying business failure, and included more granular categories of business failure.

### 6.2. Research Contributions

This research contributes to the literature of business recovery and decline by building a sequential data-based framework to predict the performance changes of organizations under risks. There are some prediction-based research in the field of business recovery and decline, but prior research either focuses on business decline (Li et al., 2016; Zhou et al., 2017) or recovery (Zhou, 2013), and none has included both of them in the predictive model. Including both types of organization performance dynamics offers managers the ability to track a portfolio of organization performance changes.

This research contributes to the literature of machine learning-based organization performance prediction by introducing the dynamic SDP-OPC framework that is capable of processing time series data. The prior literature mainly uses static models to predict business failure and business recovery (Wang and Li, 2020; Li et al., 2021). Our experiments confirmed that incorporating time series data can significantly improve the performance of the prediction model. The SDP-OPC framework also supports the analysis of mass media sentiment and including mass media sentiments as features to improve the prediction accuracy. Prior research mainly use the textual features of annual reports such as superficial tones, text emotional value and text readability to predict the delisting of pubic companies (Wang and Li, 2020; Li et al., 2021), and only few incorporate the mass media sentiment.

This research also adds to an emerging stream of research on building organization intelligence using advanced artificial intelligence-based decision-making algorithms to achieve sustainable organization performance (Galbraith and Podhorska, 2021; Suler et al., 2021). This stream of research uses various artificial intelligence algorithms and data sources, including not only traditional accounting and financial data, but also textual data, organization process data and Internet of things data to monitor organization performance at both the strategic and operational levels (Cunningham, 2021; Galbraith and Podhorska, 2021; Kovacova and Lăzăroiu, 2021; Suler et al., 2021). The insights generated from the data can be used to enable cognitive automation, one important management method that contributes to sustainable organization performance (Galbraith and Podhorska, 2021; Hopkins and Siekelova, 2021; Smith and Machova, 2021; Zvarikova et al., 2021).

This research also offers important managerial implications. Firstly, this framework can be readily used by organizations to monitor and predict their performance changes and accordingly understand their risk status. In the recent years, organizations increasingly suffer from the conditions of VUCA (volatility, uncertainty, complexity, ambiguity) (Li and Zhu, 2021), and business downturn and upturn happen more often than ever. Being able to predict the future possible business downturn and upturn can help organizations stay prepared and respond quickly by making adjustments and improvements. Except for monitoring the organization performance changes, the SDP-OPC framework also reports the feature importance, which can be used by managers as a guidance to take measures to achieve business recovery or prevent business decline. Secondly, this work is particularly relevant to investors in the China investment market. Some investors, especially foreign investors new to the China investment market, have little investment experience and lack risk analysis tools. The framework proposed in this paper provides investors an effective method to predict the risk status changes of listed companies, and enables them to make informed investment decisions. Finally, this work facilitates the supervision of financial markets. The stock market regulatory institutions can use the SDP-OPC framework to detect and warn those organizations with high probability of business decline, and provide support to those with a high probability of business recovery. Such actions will help to maintain and ensure the stability of the financial market.

### 6.3. Limitations and Future Research

This research is limited in several aspects, which offer some future opportunities. First, due to the limited computation resources, this work only focuses on listed companies in China during the period of January 1, 2017 to December 31, 2019. Future research can include a longer time period to validate the framework. Second, the original dataset is highly imbalanced. This research uses under-sampling method to deal with the problem. In future research, other solutions to the imbalanced data problem can be explored, such as the synthetic minority oversampling technique (SMOTE) combined with the Adaboost support vector machine ensemble integrated with item weighting (ADASVM-TW) (Sun et al., 2020). Finally, this research uses only news titles as sources of non-structured data. Future research can aggregate data from more sources, such as social media and mass media of other sources, to further validate the proposed framework. Given the computation resources, future research can process the entire article to calculate and include more sentiment metrics such as uncertainty and anxiety in the prediction model.

## 7. Conclusions

As the business environment is increasingly turbulent and dynamic, maintaining sustainable and robust organization performance becomes increasingly challenging. Predicting organization performance changes provides an early risk warning for managers to stay prepared for such a change. This research builds a sequential data-based prediction model to predict a portfolio of organization performance changes, including both business decline and business recovery. The model incorporates both structured and unstructured data from various sources, as well as time series data to improve the accuracy of the model. The experiments confirm that the proposed SDP-OPC framework outperforms other benchmark models. The research adds to the organization intelligence management by offering managers a practical tool to monitor organization performance changes. Future research can advance this stream of research by introducing more algorithms to process the imbalanced data and aggregating data from more sources.

## Data Availability Statement

The raw data supporting the conclusions of this article will be made available by the authors, without undue reservation.

## Author Contributions

MS contributed to the design, data acquisition, experiments and drafting of the article. XF contributed to the design and drafting of the article. SW, ZD, and YZ contributed to drafting of the article. All authors read and approved the final manuscript.

## Funding

This research is supported by the Social Sciences and Humanities Research Council of Canada (SSHRC) Grant (grant no: 435-2020-0761).

## Conflict of Interest

The authors declare that the research was conducted in the absence of any commercial or financial relationships that could be construed as a potential conflict of interest.

## Publisher's Note

All claims expressed in this article are solely those of the authors and do not necessarily represent those of their affiliated organizations, or those of the publisher, the editors and the reviewers. Any product that may be evaluated in this article, or claim that may be made by its manufacturer, is not guaranteed or endorsed by the publisher.
